# Characteristics of macrophage-like cells in acute nonarteritic anterior ischemic optic neuropathy and the normal fellow eyes on en face optical coherence tomography

**DOI:** 10.3389/fimmu.2022.1095296

**Published:** 2022-12-23

**Authors:** Wenyu Wang, Changzheng Chen, Zuohuizi Yi, Xiaoling Wang, Huijuan Luo

**Affiliations:** ^1^ Department of Ophthalmology, Renmin Hospital of Wuhan University, Wuhan, China; ^2^ Department of Ophthalmology, Yidu People’s Hospital, Yichang, China

**Keywords:** en face OCT, retina, nonarteritic anterior ischemic optic neuropathy, macrophage-like cells, inflammation

## Abstract

**Purpose:**

To quantitatively analyze macrophage-like cells (MLCs) at the vitreoretinal interface (VRI) in acute nonarteritic anterior ischemic optic neuropathy (NAION) using en face swept-source optical coherence tomography (OCT).

**Methods:**

Twenty-five acute NAION eyes and 25 normal fellow eyes from 25 patients were included in the study. MLCs were visualized in a 3 μm 6 mm×6 mm en face OCT slab above the VRI centered on the optic nerve head (ONH). After semiautomatic binarization and quantification, we evaluated the MLC density between the two groups. We also investigated the relationship between MLC density and other OCT parameters, including the increase in peripapillary retinal nerve fiber layer (RNFL) thickness and loss of vessel density (VD) in radical peripapillary capillary (RPC).

**Results:**

The MLC density in the affected eye of the ONH was highly correlated with that in the fellow eye (r=0.612, p=0.001). The MLC density significantly increased in acute NAION eyes (NAION vs. normal, 11.97 ± 6.66 vs. 9.31 ± 6.10 cell/mm^2^, p=0.028). In sectorial analysis, the increase in MLCs was mainly in the superior regions (4.13 ± 7.49 vs. 0.94 ± 5.21 cell/mm^2^, p=0.001). The VD of RPC decreased significantly in the affected eyes (NAION vs. normal, 37.54 ± 5.25 vs. 40.56 ± 4.25, p=0.016), and the loss of RPC was predominantly in the superior sectors and the temporal sectors when compared to the inferior sectors and the nasal sectors, respectively (superior vs. inferior, -3.54 ± 6.71 vs. -0.37 ± 8.07, p=0.004; temporal vs. nasal, -2.69 ± 8.72 vs. -1.22 ± 6.06, p=0.005). The loss of RPC and the increase in MLC density were greater in affected sector corresponding to the visual field (VF) defect.

**Conclision:**

MLCs located above the VRI increased significantly in acute NAION eyes, especially in subregions corresponding to VF defect, which provides clinical evidence supporting that the inflammatory response participates in the pathological process of NAION. The magnitude of the increase in MLCs corresponds to the RPC loss in the quadrant analysis.

## 1 Introduction

Nonarteritic anterior ischemic optic neuropathy (NAION) is the most common cause of sudden optic nerve-related vision loss and typically occurs in individuals over 55 years of age (1). As reported, the prevalence of NAION in the general population older than 40 was 102.87 per 100 000 people (2). The actual etiology of NAION is controversial. A crowded disk is an acknowledged risk factor (3). Additionally, there are many other systemic risk factors associated with NAION, such as hypertension, hyperlipidemia, atherosclerosis, diabetes mellitus, episodic hypotension, and obstructive sleep apnea (4–10). It is generally believed that NAION is due to acute ischemia of the optic nerve head (ONH) supplied from the posterior ciliary artery circulation (11). Repeated studies demonstrated the loss of peripapillary capillary (RPC) and retinal nerve fiber layers (RNFL) predominantly in the superior sectors from the acute to the chronic phase (12, 13). Changes in vascular and neural structures seemed to correlate with the severity of visual field (VF) defects (12–15).

Although NAION is different from inflammatory optic nerve diseases such as optic neuritis, some evidence has shown that the inflammatory response is involved in the pathological process of NAION. Studies have shown the accumulation of inflammatory cells—both intrinsic, activated microglia and extrinsic macrophages—in rat models of NAION (9). The same findings were observed in a human histological specimen (16) and primate models (17). Histopathologic studies of human specimens found that ischemic optic nerve lesions were initially acellular and later showed macrophage infiltration (15). Other histopathologic findings also suggested the destruction of the blood retinal barrier. Some studies have shown that systemic macrophages, rather than intrinsic microglia, are involved in eliminating degenerating myelin from fragmenting axons (18). This could be a potential therapeutic target to protect the optic nerve and reduce the loss of retinal ganglion cells.

Recently, some groups have reported that macrophage-like cells (MLCs) located at the inner limiting membrane (ILM) were successfully observed in live human retina using adaptive optics optical coherence tomography (AO-OCT) and clinically used optical coherence tomography (OCT) (19–22). MLCs increased in many retinal diseases, including diabetic retinopathy (19), retinal vein occlusion (23, 24), uveitis (25) and central retinal artery occlusion (26). A study based on animal models suggested that MLCs are a potential biomarker for inflammation during retinal vascular disease (27).

Although the observation of inflammatory cells in optic nerve axons is difficult with a clinical OCT device, investigation of peripapillary macrophages located above the vitreous retinal interface can provide new insights into the role of inflammation in NAION. To the best of our knowledge, this study is the first to investigate MLCs in the ONH region in NAION eyes with en face OCT. We aimed to illuminate the number and distribution of macrophage-like cells near the optic disc and the relationship between the cells and the changes in other optical coherence tomography angiography (OCTA) parameters. This study will provide clinical evidence for further understanding the pathological process of NAION.

## 2 Materials and methods

### 2.1 Subjects

This observational cross-sectional study included 50 eyes of 25 patients with unilateral acute NAION. (Symptoms occurred within two weeks.) The unaffected normal fellow eyes were used as a control group. The study was approved by the Institutional Review Board of the Renmin Hospital of Wuhan University (WDRY2021-k162) and conducted in accordance with the tenets of the Declaration of Helsinki, and informed consent was obtained from all participants. A diagnosis of NAION was made based on patients’ symptoms, clinical examination and imaging, which included visual acuity, VF, intraocular pressure (IOP), fundus fluorescein angiography (FFA), visual evoked potential (VEP), and color fundus photography. MRI was also performed to rule out papilloedema induced by neurological diseases. The erythrocyte sedimentation rate and C-reactive protein tests were performed to exclude arteritic ischemic optic neuropathy in all subjects. Patients with a sudden decrease in visual acuity in one eye with a clinical diagnosis of acute NAION and with normal fellow eyes were included in the study. Inclusion criteria (1): monocular acute visual loss with optic disc edema; (2) typical horizontal visual field defect; (3) no signs of giant cell arteritis including elevated erythrocyte sedimentation rate and C-reactive protein levels; (4) no signs of neurological diseases Exclusion criteria: (1) visual loss duration more than 2 weeks; (2) subjects received any treatment including systematic administration of steroids; (3) subjects with other ocular diseases (glaucoma, uveitis, pathologic myopia, central serous chorioretinopathy, optic neuritis, etc.); (4) OCTA images with poor quality (scan quality <5 or obvious motion artifact); (5) the fellow eye was diagnosed with NAION; (6) subjects with refractive error > 3.0 diopters (D) or <-6.0 D.

### 2.2 Image acquisition and processing

**Figure 1:**
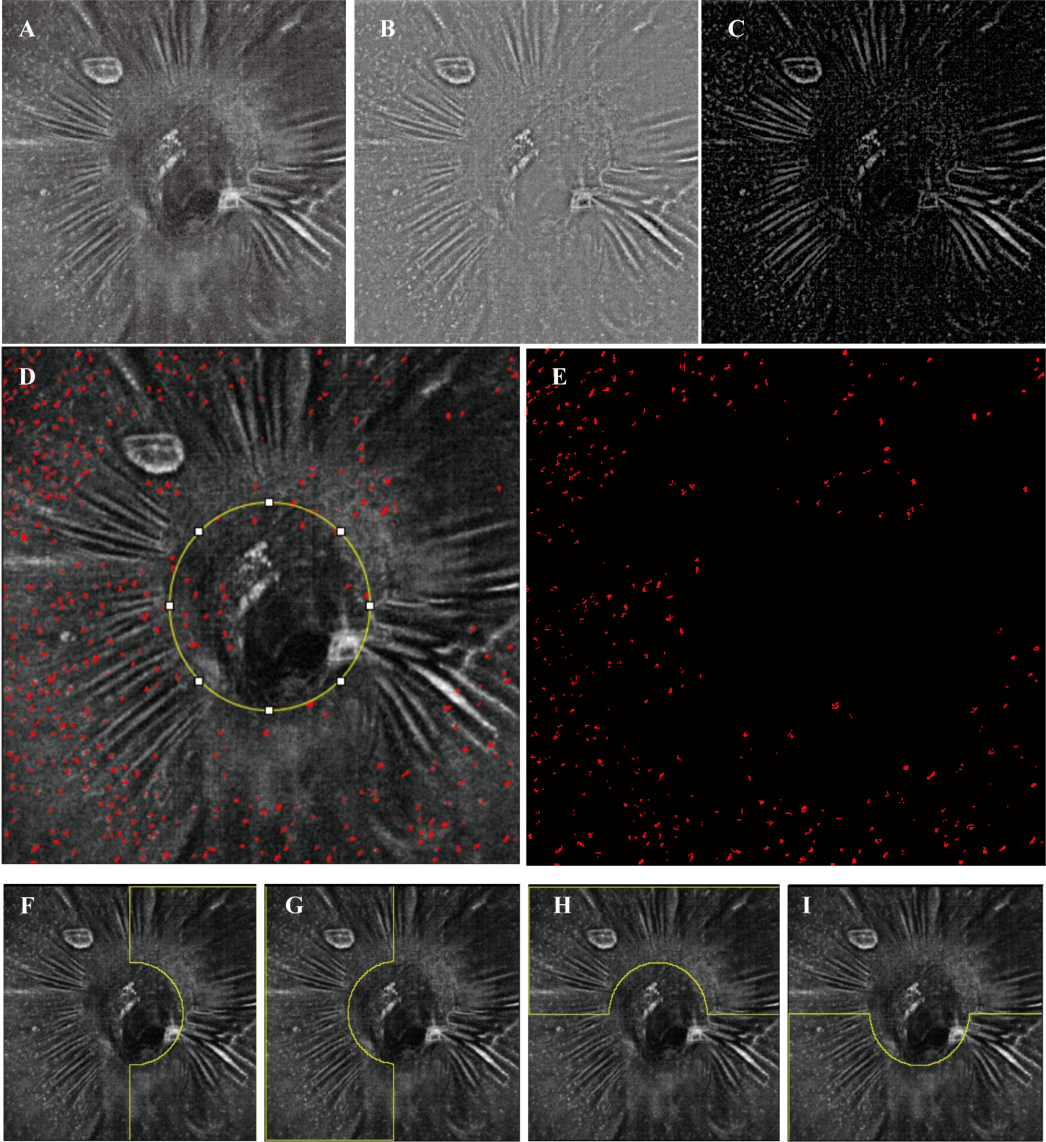
Examples of image processing and division of subregions. **(A)** Registered and averaged en face OCT slab of MLCs. **(B, C)** Background-subtracted slab and MLC signal from **(B)** was compensated by the blurred MLC layer OCT and RNFL layer (not shown). **(D)** Merged image of the MLC (red spots) extracted image and **(A)**. The yellow circle in **(D)** outlines the optic disc. **(E)** The final image for quantitation of MLCs. MLCs in the yellow circle in **(D)** are excluded. **(F–I)** Examples (the left affected eye from an acute NAION patient) of the manually divided regions of temporal, nasal, superior and inferior.

OCTA and en face OCT images covering an area of 6 mm × 6 mm centered on the optic disc were acquired at three sessions using a commercial spectral domain OCT System with a scan rate of 70,000 A-scans per second (Avanti RTVue-XR; Optovue, Fremont, CA, USA). The peripapillary RNFL thickness was also evaluated at the peripapillary region and in 4 sectors using ONH analysis software (Angio DiscVue). Each en face OCT and OCTA image was composed of a merged X-fast and Y-fast volumetric raster scan. To reduce artifacts and increase the credibility and repeatability of the results, we repeated images (range 5–7 repeats) at the same location in the retina. For imaging the MLCs on the ILM surface, a 3-μm OCT-R slab located above the ILM was used, which is consistent with previous studies (19, 20). OCTA slabs for the full retina were segmented from the ILM to 9 μm below the outer plexiform layer. Image registration and averaging were performed on the en face OCT slabs and OCTA images to enhance visualization of MLCs and reduce the artifact using the Register Virtual Stack Slices and plug-in on ImageJ (ImageJ, US National Institutes of Health, Bethesda, MD, USA). Briefly, the registered and averaged images underwent noise reduction to remove vessel artifacts and were binarized to extract discrete cell shapes. The specific methods are consistent with previous studies (19, 20). The resulting image was first converted to an 8-bit image and then binarized. Examples of image processing are shown in **Figure 1**. Because MLCs located on the ONH were hard to distinguish from the artifacts, they were eliminated in quantitation (yellow circle in **Figure 1D**). The Analyze Particles tool was used to analyze the number, area, and percentage of MLCs.

**Figure 2:**
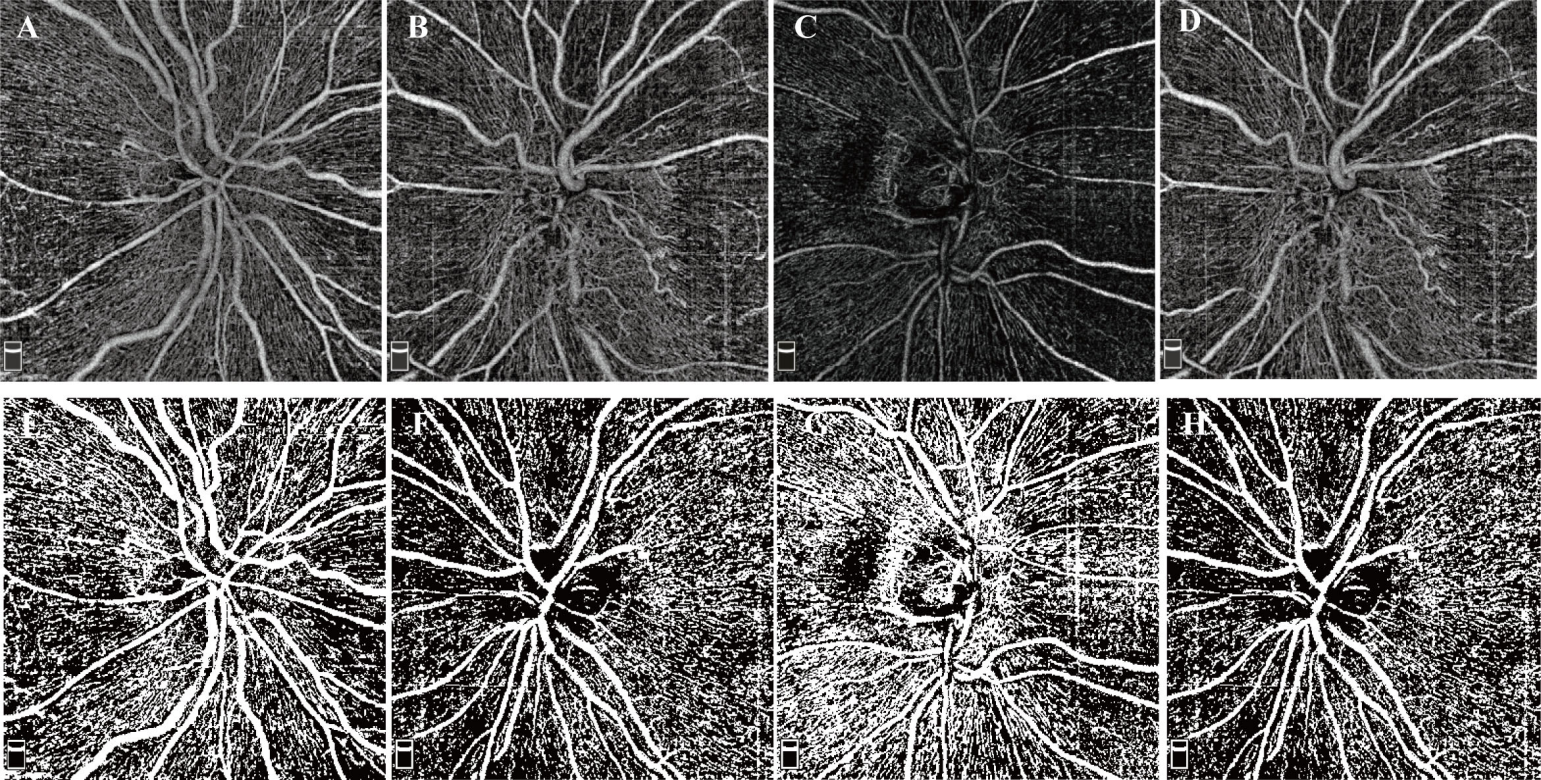
Examples of RPC OCTA images and binarized images. **(A–D)** RPC OCTA images of four eyes from two NAION patients. **(A)** OCTA image from the normal right eye of patient S. **(B)** OCTA image from the affected left eye of patient S. **(C)** OCTA image from the normal right eye of patient P. **(D)** OCTA image from the affected left eye of patient P. **(E-I)** Examples after thresholding procession. **(E–I)** correspond to **(A–D)**, respectively.

The OCTA slabs for PPC were automatically generated by the built-in software. The registered and averaged RPC images were binarized with Huang thresholding (19) (examples shown in **Figure 2**). The vessel density (VD) was determined as the percentage of area occupied by blood vessels on the PRC images.

The peripapillary region was further divided into two parts according to two methods: 1) the superior region and the inferior region (examples shown in **Figure 1H, I**); 2) the nasal region and temporal region (examples shown in **Figure 1F, G**). The MLCS density and VD of RPC were measured in these subregions.

### 2.3 Statistic

We used SPSS Statistics (version: 26.0; IBM Corp., Armonk, NY, USA). Continuous variables with a normal distribution are presented as the mean ± standard deviation (SD). The Shapiro−Wilk test was used to test the normality of the data. And levene’s test was used for equality of variances. In the analysis of MLC density, VD of RPC, and RNFL thickness, we performed a paired t test for parametric data. For data whose difference did not conform to a normal distribution, we used the Wilcoxon signed-rank test. In sectorial analysis, we used two-ways repeated measures analysis of variance (ANOVA). Pearson’s correlation coefficient was used to evaluate the linear correlation in MLC density between the affected eye and the fellow eye.

## 3 Results

A total of 25 patients were included in the current study. Twenty-five acute NAION eyes and 25 normal fellow eyes were evaluated. The time interval from NIAON onset to en face OCT acquisition was 9.56 ± 4.30 days (mean± standard deviation). The demographic characteristics, best corrected visual acuity (BCVA), the results of automated perimetry and risk factors for NAION are summarized in **Table 1**.

**Table 1:** Demographic characteristics of subjects.

	NAION eyes	Fellow eyes	P
Subjects, n	25		–
Age, mean±SD	56.76±8.64		–
Sex (female), n (%)	7 (28)		–
BCVA (logMAR)	0.20±0.18	0.13±0.12	P*=0.045
Refractive error (D), mean±SD	-0.1±1.0=19	-0.05±1.08	P**^†^** =0.678
Automated perimetry MD, dB	-13.59±2.63	-2.14±1.44	P**^†^** <0.001
Smoking, n (%)	6 (24)		–
Hypertension, n (%)	4 (16)		–
Diabetes mellitus, n (%)	5 (20)		–
Hypercholesterolemia, n(%)	6 (24)		–
Sleep apnea, n(%)	2 (8)		–
No risk factors, n (%)	5 (20)		–

^*^ Statistical significance was calculated with the Wilcoxon Signed Rank Test.

**^†^** Statistical significance was calculated with the Student’s t test.

BCVA, best corrected visual acuity; MD, mean deviation.

**Figure 3:**
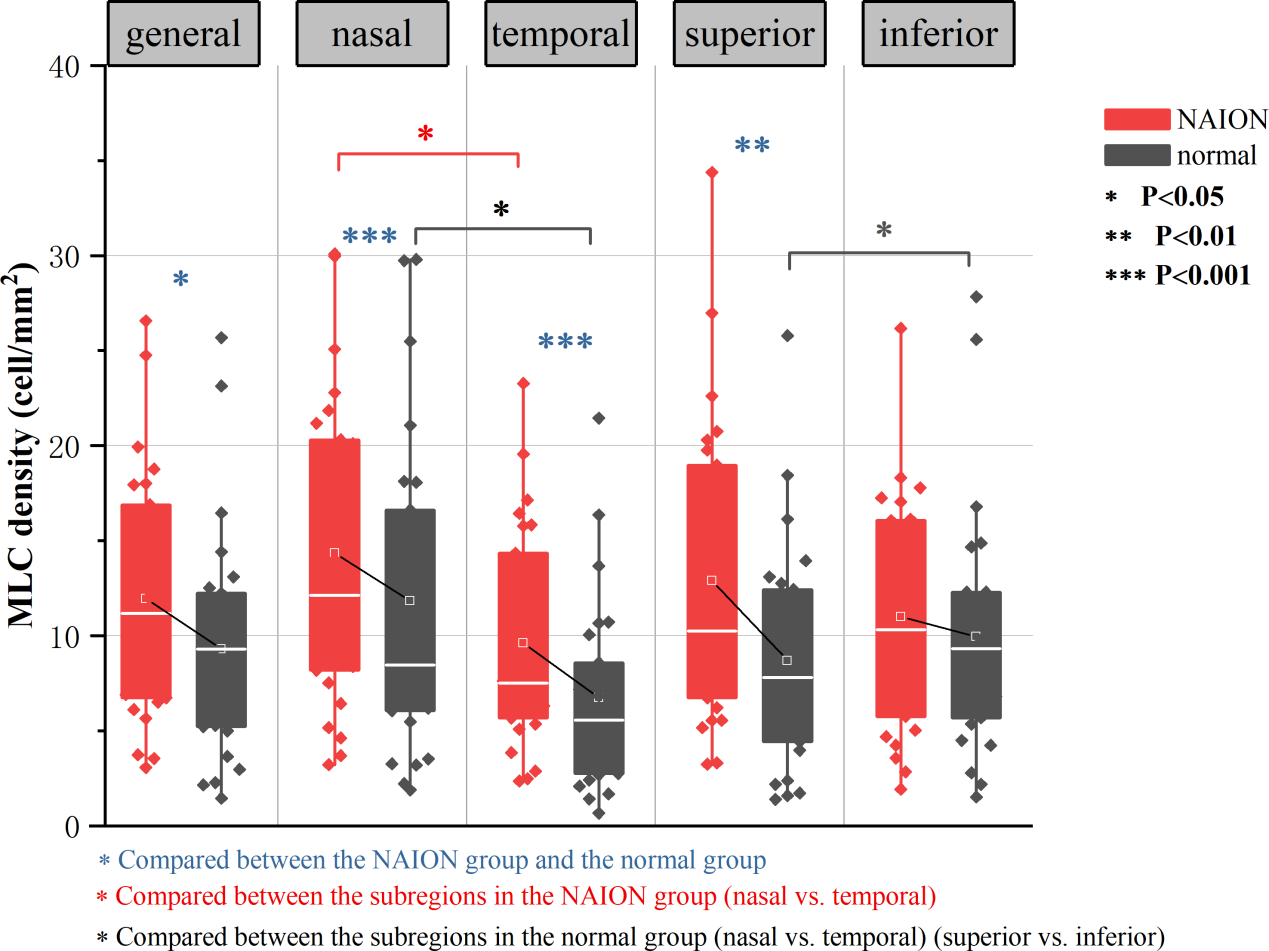
Result of two-ways repeated measures ANOVA in analyzing the effect of diasease and subregions in MLC density. Both the NAION group and the control group showed significantly increased MLC density in the nasal region than in the temporal region (p=0.026). In normal group, there were more MLCs in the inferior than in the superior (p=0.021). MLC density was higher in the NAION group (p=0.028), In horizontal sector analysis, MLC density increased in NAION eyes especially in the superior (p=0.008) when compared to the inferior subregion (p=0.315). In vertical sector analysis, MLC density increased significantly in NAION eyes in both temporal and nasal subregions (p=0.001).

**Figure 4:**
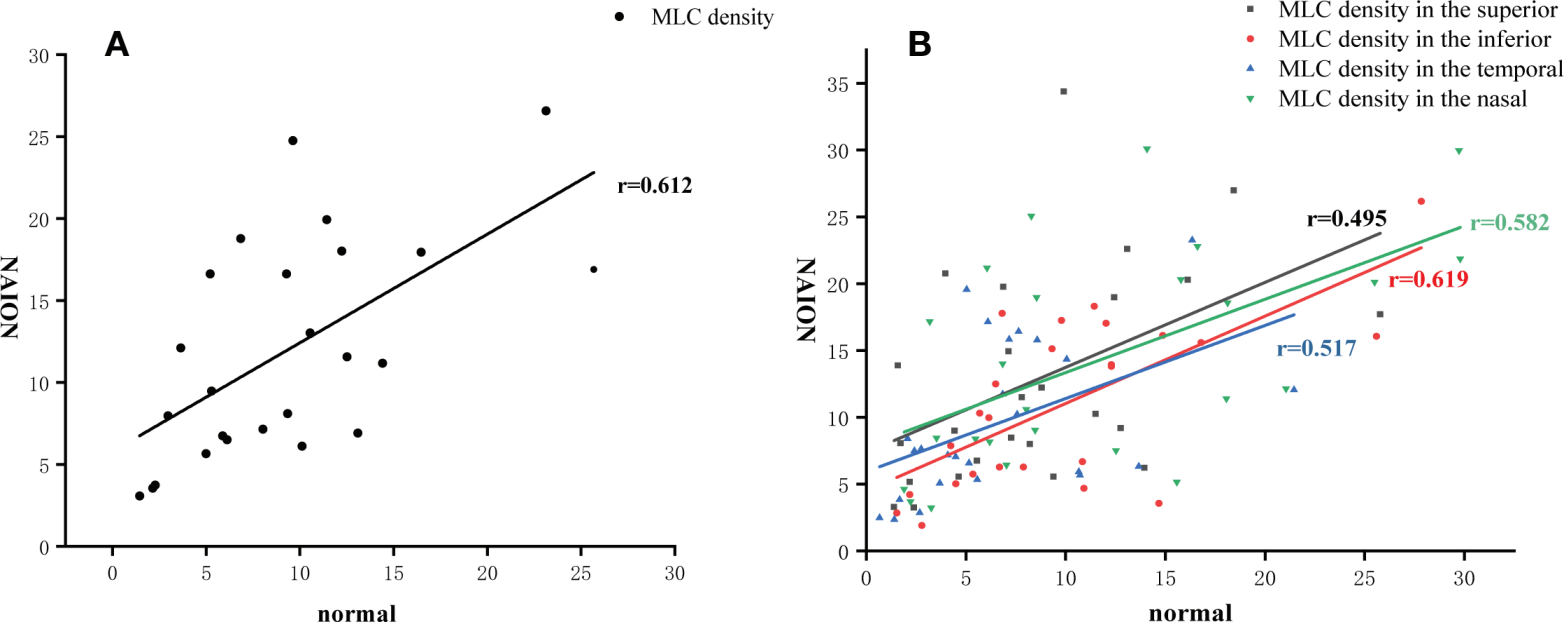
MLC density in the affected eye correlated to the fellow eye. **(A)** The positive correlation of MLC density in the total area between the NAION eyes and the fellow eyes. **(B)** The positive correlation of MLC density in four subregions between the NAION eyes and the fellow eyes.

MLCs distributed unevenly around the ONH in both NIAON eyes and normal eyes. In two-ways repeated measures ANOVA, c a higher MLC density in the nasal distribution was found in both NAION eyes (9.62 ± 5.74 vs. 14.35 ± 8.13, p=0.026) and normal eyes (6.74 ± 4.99 vs. 11.83 ± 8.32, p=0.026) when comparing to the temporal. Additionally, more MLCs in the control group were found in the inferior than in the superior region (9.95 ± 6.50 vs. 8.69 ± 5.92, p=0.021). In the affected group, the MLC density was slightly higher in the superior region, and no difference was found between the inferior and superior regions (11.01 ± 6.27 vs. 12.91 ± 7.97, p=0.086) (**Figure 3**). The MLC count and density showed large variation between individuals, but those values for NAION eyes compared to fellow eyes showed a positive correlation (r=0.612, p=0.001). In addition, the same results were found in the nasal (r=0.582, p=0.002), temporal (r=0.517, p=0.008), superior (r=0.495, p=0.012) and inferior (r=0.619, p=0.001) subregions (**Figure 4**).

**Figure 5:**
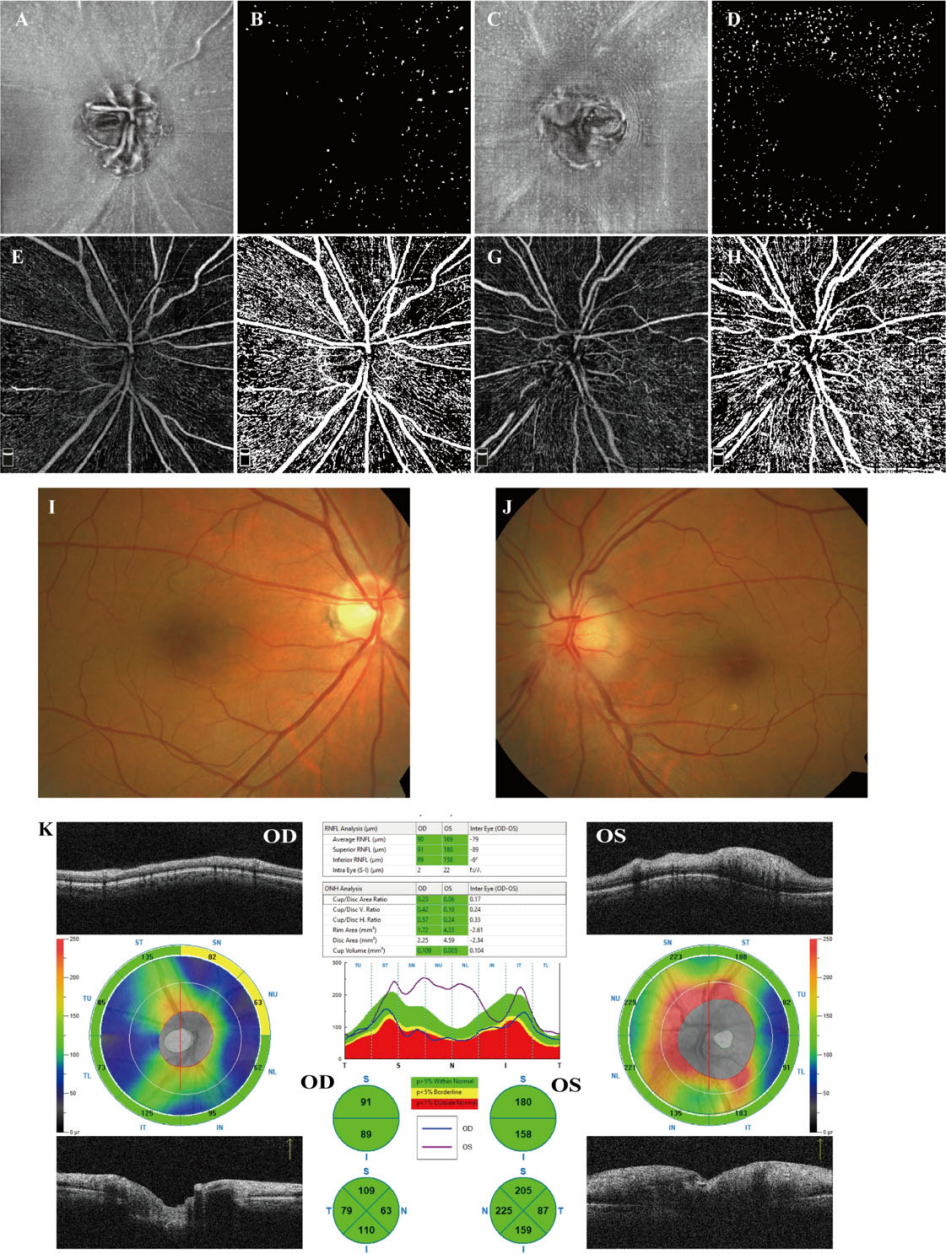
Examples from a 58-year-old male patient who suffered from a sudden visual decrease in the left eye within 2 weeks and was diagnosed with acute NAION. **(A, B)** en face OCT slab of MLCs from the normal right eye and its binarized image for MLC counting. **(C, D)** en face OCT slab of MLCs from the affected left eye and its binarized image for MLC counting. Compared to **(B)**, cells in **(C)** increased significantly, particularly in the superior sector. **(B, D)** More MLCs are found in the nasal ONH in both affected eye and fellow eye. **(E, F)** OCTA image of RPC from the right eye and its binarized image adjusted by Huang thresholding for VD measurement. **(G, H)** OCTA image of RPC from the left eye and its binarized image for VD measurement. In the affected eye, the loss of RPC was mainly identified as superior. **(I, J)** Color fundus photograph showing papilledema and retinal vascular tortuosity in the left eye. **(K)** The peripapillary RNFL thickness analysis illustrated that RNFL thickness increased significantly in the affected eyes, particularly in the superior, inferior and nasal sectors.

**Figure 6:**
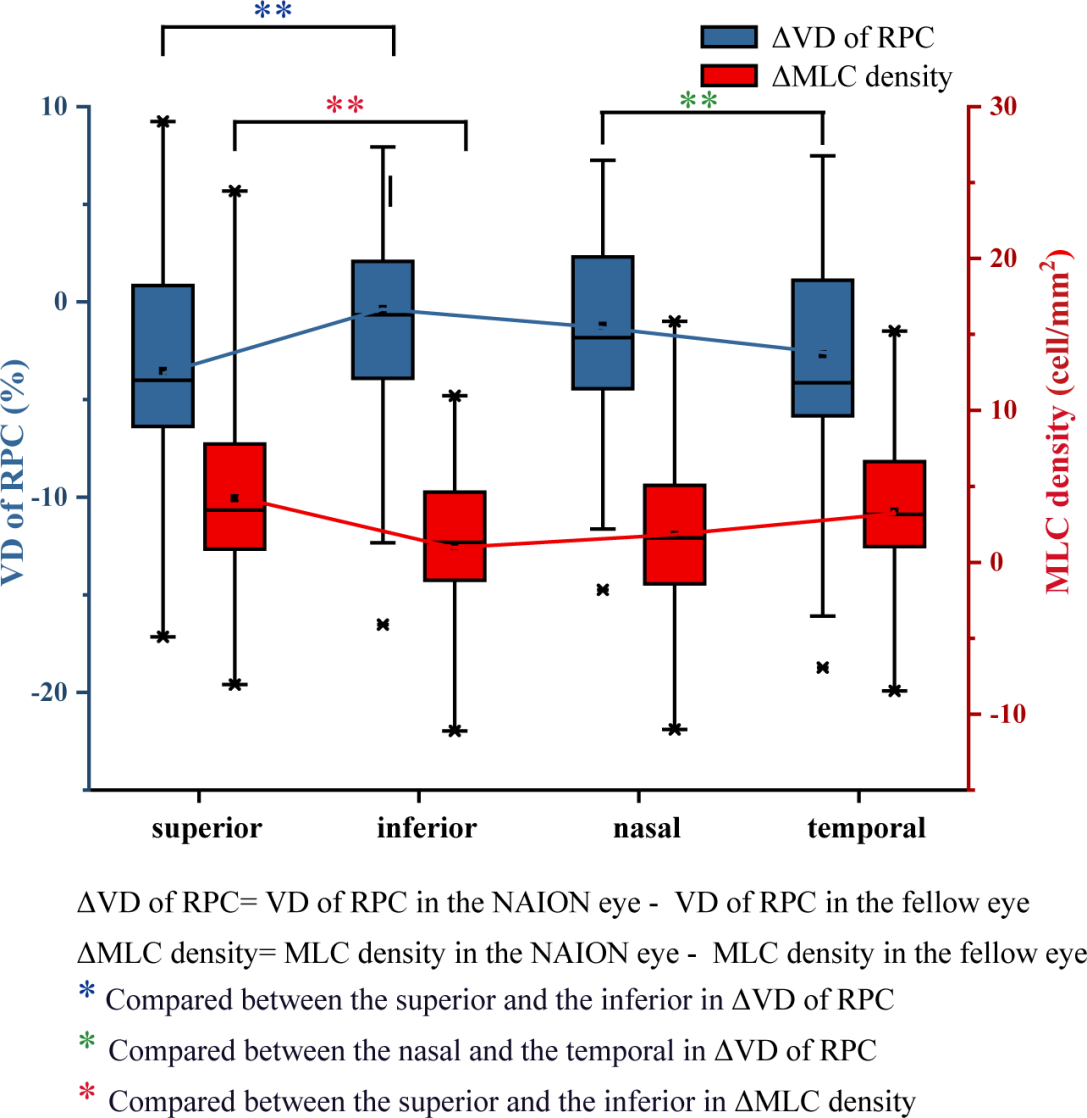
Differences in VD of RPC and the MLC density between the NAION group and the control group. Compared with the fellow eye, the decrease in VD of RPC in the superior eye was significantly greater than that in the inferior eye. The increase in the MLC density in the superior and temporal regions was greater than that in the inferior and nasal regions.

Despite the similar distribution characteristic, MLC density increased significantly in the acute NAION eyes (NAION vs. normal, 11.97 ± 6.66 vs. 9.31 ± 6.10 cell/mm^2^, p=0.028) (examples shown in **Figure 5**). Results of paired sample T test between two groups were shown in **Table 2**. In two-ways repeated measures ANOVA, we found that the increase of MLC density was significant in superior, nasal and temporal subregions (NAION vs. normal, p=0.008, p=0.001, p=0.001, respectively). No statistical difference was found between the two groups in inferior region (NAION vs. normal, 11.01 ± 6.27 vs. 9.95 ± 6.50, p=0.315). In NAION group, the increase in the MLC density was uneven among the subregions. The amount of change in the superior subregions was greater than that in the inferior sides (4.13 ± 7.49 vs. 0.94 ± 5.21 cell/mm^2^, p=0.001) (**Figure 6**). The same result was not found in the comparison between the temporal sector and the nasal sector (3.23 ± 5.69 vs. 1.68 ± 7.87 cell/mm^2^, p=0.301). In addition, the VD of RPC decreased significantly in the affected eyes (NAION vs. normal, 37.54 ± 5.25 vs. 40.56 ± 4.25, p=0.016). In the sectorial analysis, we found that the VD of RPC showed a reduction predominantly in the superior sectors and the temporal sectors when compared to the inferior sectors and the nasal sectors, respectively (superior vs. inferior, -3.54 ± 6.71 vs. -0.37 ± 8.07, p=0.004; temporal vs. nasal, -2.69 ± 8.72 vs. -1.22 ± 6.06, p=0.005) (**Figure 5**, **6**). The loss of RPC seems to correspond to the increase in the MLC density. Compared with the fellow eye, RNFL thickness increased significantly in four sectors (**Table 2**). There were no significant differences in the increase in RNFL thickness among the four sectors (p=0.414).

**Table 2:** Parameters of NAION eyes and the control eyes.

	NAION eyes	Fellow eyes	P
MLC density (cell/mm2)	11.97 ± 6.66	9.31 ± 6.10	**P^†^=0.028**
Superior	12.91 ± 7.97	8.69 ± 5.92	**P^†^=0.008**
Inferior	11.01 ± 6.27	9.95 ± 6.50	P**^†^** =0.315
Temporal	9.62 ± 5.74	6.74 ± 4.99	**P^†^=0.016**
Nasal	14.35 ± 8.13	11.83 ± 8.32	P**^†^** =0.115
VD of RPC (%)	37.54 ± 5.25	40.56 ± 4.25	**P^†^=0.018**
Superior	36.72 ± 5.28	40.26 ± 4.75	**P^†^=0.016**
Inferior	38.77 ± 8.09	39.14 ± 4.72	P^*^ =0.511
Temporal	39.97 ± 7.83	42.66 ± 4.90	P^*^ =0.052
Nasal	35.52 ± 5.73	36.74 ± 4.83	P**^†^** =0.333
RNFL thickness (μm)	180.54 ± 53.26	108.25 ± 15.27	P^*^<0.001
Superior	208.18 ± 69.81	135.82 ± 21.24	P^*^=0.002
Inferior	202.53 ± 65.91	143.65 ± 29.42	P^*^=0.001
Temporal	155.41 ± 89.90	78.29 ± 12.16	P^*^<0.001
Nasal	156.06 ± 58.02	75.24 ± 10.98	**P^†^=0.001**

Date are presented as mean ± SD. MLC, macrophage-like cells; VD, vessel density; RNFL, retinal nerve fiber layer; RPC, peripapillary radical capillary.

^*^ Statistical significance was calculated with the Wilcoxon Signed Rank Test.

**^†^** Statistical significance was calculated with the Student’s t test. The meaning of the bold values is statistical difference (P < 0.05).

We further investigated the MLC density, VD of RPC and RNFL thickness and their difference between bilateral eyes in subregions according to the VF defect. Most patients (n=21) had inferior VF defect with superior ONH affected and others had superior VF defect with inferior ONH affected. Compared with unaffected regions, significantly increased MLC density was found in affected regions. There was a statistical difference in ΔMLC density (MLC density in NAION eye minus MLC density in fellow normal eye) and ΔVD of RPC (VD of RPC in NAION eye minus VD of RPC in fellow normal eye) between the affected and unaffected subregions. The results are shown in **Table 3**.

**Table 3:** Results of MLC density, VD of RPC and RNFL thickness in sectorial analysis.

	Affected subregion	Unaffected subregion	P
MLC density (cell/mm2)	13.49 ± 7.93	10.76 ± 6.39	P^*^=0.009
ΔMLC density (cell/mm2)	4.18 ± 6.79	1.09 ± 5.86	P^*^=0.004
VD of RPC (%)	36.67 ± 5.64	38.81 ± 7.83	P^*^ =0.061^*^
ΔVD of RPC (%)	-3.54 ± 6.71	-0.64 ± 7.86	P^*^=0.014^*^
RNFL thickness (μm)	191.18 ± 74.91	172.41 ± 92.93	P**^†^** =0.38
ΔRNFL thickness (μm)	75.00 ± 82.98	58.25 ± 72.36	P**^†^** =0.16

MLC, macrophage-like cells; VD, vessel density; RNFL, retinal nerve fiber layer; RPC, peripapillary radical capillary; ΔMLC density= MLC density in normal fellow eye – MLC density in NAION eye; ΔVD of RPC= VD of RPC in normal fellow eye – VD of RPC in NAION eye; ΔRNFL thickness= RNFL thickness in normal fellow eye – RNFL thickness in NAION eye.

^*^ Statistical significance was calculated with the Wilcoxon Signed Rank Test.

**^†^** Statistical significance was calculated with the Student’s t test. The meaning of the bold values is statistical difference (P < 0.05).

There was no correlation among the decrease in VD of RPC, the increase in RNFL thickness and the increase in MLC density after controlling the age, sex and disease duration

## Discussion

MLCs around the ONH characterized by en face OCT were detected in the current study. To the best of our knowledge, this is the first study to investigate macrophage-like cells *in vivo* with acute NAION. We explored the cell distribution characteristics around the ONH, the uneven increase in MLCs and the correlation among the increase in cell density, the decrease in VD of RPC and the VF defect.

In our study, we found that whether in normal eyes or in NAION eyes, the distribution of cells around the optic disc followed a certain pattern. Significantly higher cell density was found in the nasal sectors than in the temporal sectors. In the normal eyes, more MLCs located in the inferior sectors than in the superior sectors. Due to the greater increase in the superior sectors, the same pattern was not found in the affected group. Repeated studies have demonstrated that MLCs located unevenly on the vitreoretinal interface (VRI). A previous study found that the cell density was higher at the macular temporal than at the ONH (20). In our observation of RVO patients, the en face OCT images centered on the macular illustrated that most MLCs in the normal eyes were found in the perifovea region, and only a few cells were found within 3 mm of the fovea. A recent study in a mouse model that combined OCT and confocal immunofluorescence found that the cells located above the membrane are heterogeneous (27). Cells with different identities are not evenly distributed on the VRI. Wild field en face OCT is needed to explore the distribution characteristics of human retina MLCs. Another interesting finding is that although individual differences in cell numbers were evident, there was a positive correlation between the number of cells in both eyes. This result was true in all subregions around the ONH, which was consistent with our previous findings (24). It would be better to design a paired experiment in the MLC investigation to eliminate differences between individuals.

We found that MLC density in the ONH region increased significantly in the NAION group, especially in the affected subregions corresponding to the VF defect. According to previous studies, inflammation plays a role in the pathological process of NAION. Researchers found that the neutrophil-to-lymphocyte ratio and some cytokines, chemokines, and growth factors in the peripheral blood of NAION patients were significantly higher than those of the controls (28) (29). The inflammatory status of patients with NAION suggests that the onset and progression of the disease may be related to inflammation. The local inflammatory response was further studied in animal models. Similar to other central nervous system infarct and spinal cord injury models, NAION and sudden (optic nerve) ON ischemia result in early cytokine-mediated changes followed by sequential inflammatory cellular activation and infiltration. After the induction of ON ischemia, the breakdown of the blood-optic nerve barrier occurs within hours (30–32), followed by the recruitment of extrinsic macrophages and the activation of microglia in the ischemic region (16, 33). A study based on the primate model showed that the activation of the inflammatory response may be related to axon loss (16). Histopathologic studies on human NAION specimens showed that the macrophages infiltrated the affected optic nerve (15). However, the function of inflammation is still controversial. Macrophage activity can be either neurodegenerative and/or neuroprotective in ischemic stroke (34). Early methylprednisolone treatment and granulocyte colony-stimulating factor can be effective in reducing macrophage recruitment and ON vascular permeability and resulting in more survival retinal ganglion cells (30, 35). In the current study, we found an increase in MLCs in living NAION patients using a clinical OCT device, which was consistent with previous studies. MLC on the VRI was observed in several retinal diseases, including diabetic retinopathy (19), retinal vein occlusion (23), uveitis (25), retinal artery occlusion (24, 26) and posterior vitreous detachment (36). Some researchers considered the MLCs above the VRI as potential biomarkers of inflammation during retinal vascular disease (27). Our findings provide clinical insights into the involvement of inflammation in the postischemic pathological process in NAION. However, we did not determine the definite identity and function of these cells in the cross-sessional clinical study. Further studies on animal models combining clinical OCT and confocal immunofluorescence are needed to investigate the exact mechanism.

Acute ischemia of the optic nerve is always followed by severe swelling of the peripapillary RNFL, loss of retinal ganglion cells and loss of the peripapillary RPC, which has been demonstrated by clinical studies based on OCT and OCTA and experimental studies based on animal models (12, 13, 37–44). A few studies found that the change in the RNFL and RPC was predominantly in superior sectors, corresponding to visual field defects (14). In sectorial analysis, we also found that compared with the fellow eye, the decrease in VD of RPC was more significant in the superior sector and the temporal sector. Analysis based on VF affected hemisphere and unaffected hemisphere, the loss in VD of RPC was more in the affected subregions, relating to the VF defect. We speculated that the recruitment of macrophages and the inflammatory response are more severe in the injured area. There was no statistical difference in RNFL thickness between the affected hemisphere and unaffected hemisphere. The mean disease duration was 9.56 days in our study. Previous study demonstrated that in acute NAION, initially the involved part of the disc has edema, followed by the entire disc showing generalized edema in several days (9). Because few patients in very early acute stage were included, the difference were not found in sector analysis of RNFL thickness.

The study is insufficient to draw the exact relationship among the decrease in VD, the loss of RNFL and the aggregation of MLCs. And no correlation was found even after adjusting for patients’ age and disease duration. In NAION eyes, the edema of ONH occurred in the acute stage and the atrophy developed after two months from onset (9, 12). RPC was also gradually lost during this process. Although the NAION eyes showed decreased VD of RPC in the early stage, some study found greater loss of RPC in the chronic stage (42). Considering that during disease duration the RNFL thickness and VD of RPC were changing, it was explainable that no correlation conclusion was drawn in the current cross-sectional study. And the sample size also needs to be further expanded. In addition, follow-up of NAION patients is required to explore the changes in MLCs with the prolongation of the disease course and find out the relationship between the aggregation of MLCs and the loss of RNFL and RPC.

There are some limitations in our study. First, because of the imaging technology, it was difficult to use blinding in processing the MLC images, and it may have some effect on a few images needing manual correction. Second, to cover a larger area, the HD 6 × 6 mm image on the ONH was used in the study. Because the VD of RPC cannot be calculated in the built-in software in the OCT device, the VD in our study was calculated by ImageJ with thresholding strategy used in a previous study. Different methods may influence the values of VD measurement. However, the characteristics of VD in the study are similar to those in other NAION studies. Last but not least, the study did not demonstrate the origin and function of the MLCs on VRI. We only included acute and treatment-naïve subjects. Further follow-up studies may focus on the response to steroids, the behavior of MLCs in the atrophic stage and the impact on visual outcomes.

In general, a significantly increasing density of MLCs on the ILM was found in acute NAION eyes. We also found a potential correlation between the increasing number of MLCs and the loss of RPC. The investigation of MLCs provided insights from clinical studies that inflammation takes part in disease progression and may be helpful for understanding the pathophysiology and for finding therapeutic targets for neuroprotection in NAION. Last but not least, the observation of MLCs is a simple and noninvasive method to evaluate the severity of the inflammatory response in daily clinical practice. As an imaging biomarker, MLCs might contribute to estimating the prognosis of NAION and guiding treatment in the clinic.

## Data availability statement

The raw data supporting the conclusions of this article will be made available by the authors, without undue reservation.

## Ethics statement

The studies involving human participants were reviewed and approved by The Institutional Review Board of the Renmin Hospital of Wuhan University (WDRY2021-k162). The patients/participants provided their written informed consent to participate in this study.

## Author contributions

CC provided guidance and overall direction throughout the study design, paper writing and revision. WW handled the literature review, data collection, data analysis and interpretation, and manuscript writing. ZY provided expertise on data collection and review the manuscript. XW collected data and reviewed the manuscript. HL collected data and provided guidance on writing. All authors contributed to the article and approved the submitted version.
